# Address

**Published:** 1869-05

**Authors:** A. T. Keightly

**Affiliations:** President of the Wabash Dental Society


### DENTAL REGISTER.

VOL. XXIII.] MAY, 1869. [No. 5

### Original Communications.

### ADDRESS,

BY DR. A. T. KEIGHTLY,

President of the Wabash Dental Society, delivered March 24, 1869.

Permit me to return to you my sincere thanks for the honor you have conferred upon me in selecting me to preside over your deliberations. It is more than an expression of mere words to say that presiding over such a body as I see before me is an honor, of which any presiding officer might be proud, although I see around me features I have never seen before. I expect that some of you may differ with me in many respects. I can not but feel that we are not strangers; are we not practitioners of the same profession and citizens of the same country, which ought to bind us together in a common destiny? Is not their honor our honor, their glory our liberty? Does not the benificence of the profession and government reflect the wisdom and happiness of all? Are we not all children of one mother, who opens her arms and ought to forget our follies, and, pardoning our crimes, enfolds all to her warm and loving heart. Those of the same profession and country ought not to be strangers. Let us, then, fellow practitioners, unite with one heart and mind.

Resorting to social intercourse, and harmony and affection may abound: still one thing more; a wise and wholesome law, which should restrain men from annoying one another, and protect industry and improvements, and shall not take from the mouth of labor the bread it has earned, let it be the bright constellation which shall protect all alike. I have full faith and confidence in this species of legislation. It is successful elsewhere, why can not it be here. In Europe and throughout her provinces, legislative enactments to regulate the practice of medicine has quite driven quackery from that field, and have taught the public to hold their educated practitioners in the highest esteem. Let the law set up a standard. Let it be denominated fraud by the law of procuring money by false pretenses. Then how long would quackery be able to hold up its head.

I am oppressed by the presence in which I stand in the midst of this city, where its handsome church spires point heavenward, and where religion seeks to soften the aspirations of life; here the luxurious homes, where culture and refinement gives charms to wealth; here the genius of law and social order presides, and that spirit of contentment which makes us a free and happy people, when I recall the past and compare it with the present. We should bow with reverence before the beneficence of that government which has fostered this wonderful development, and leaves in its hand the entire regulation of all its domestic and local affairs, and if we seek the power of the State we must remember that all power not delegated nor prohibited is reserved to the people, and to them we must look to sustain us. The Dentist must unfurl the banner of his profession and plant it upon the Constitution of his State, and claim for his profession the same respect that is awarded to others through scientific attainments and education. "Then thy virtues shall truth proclaim," placing them on high in thy sacred book, where for ages the educated Dentists will look. Then let us be ambitious to attain skill by education, still following our banner until all housed in the mansion of wisdom, to go out no more forever, and if this shall be our motto we may breast the dark storm, the red bolt defying, our wing on the wind, our eye on the sun, we will swerve not a hair, but bear onward right on. But even at this advanced and enlightened age of the world there are few things, if any, that we thoroughly understand. We see a bright flash in the storm-driven cloud and call it lightning; deep and successive peals from the artillery of the sky reverberate along the awe-stricken hills and we say that it is thunder; man is born and lives in mystery; an invisible power withdraws the vital spark; a mass of clay, he slumbers in the tomb, and crumbles to dust, and we know not how or why; give the mind nothing to do but drink in (without an effort) the beauties and the truth of nature. The ennobling pleasures of an intellectual being are the fruits of action. Let it be ours to help hasten the time when quackery shall be banished from our profession. I do not seek to avoid the difficulties that cluster around the fall of man. The day is coming when all will be made plain; then let us despise not labor, for God is its author, and a constant worker; in coming time you may expect to see him as he is; man's monument is the work he has done in this world of ours; industry is the parent of wealth in a country like this; industry will put on her garments and rest in the palace her hands and mind have built; we are here for advancement in wisdom, for we know that when the mind becomes impressed with the belief that it knows all, it ceases to advance, and perhaps the mystery of this life never will entirely be unveiled, notwithstanding the future successive revelations of science, for, even in the wisdom of God, its author is a mystery unfathomed and unlimited. Societies have been organized in every locality; they meet fraternally here, learn to value and esteem each other, and we feel that we belong to a living profession deriving light and strength. In all your deliberations, I trust you will be actuated by the spirit of charity and fraternal sympathy, and render still more glorious the name of the Association which sprang from and can only exist in a union of hearts. You will now enter upon the duties of the Association.

